# Impulsivity, Sensation Seeking, and Risk-Taking Behaviors among HIV-Positive and HIV-Negative Heroin Dependent Persons

**DOI:** 10.1155/2016/5323256

**Published:** 2016-03-08

**Authors:** Koosha Paydary, Somayeh Mahin Torabi, SeyedAhmad SeyedAlinaghi, Mehri Noori, Alireza Noroozi, Sara Ameri, Hamed Ekhtiari

**Affiliations:** ^1^Iranian Research Center for HIV/AIDS (IRCHA), Iranian Institute for Reduction of High-Risk Behaviors, Tehran University of Medical Sciences (TUMS), Tehran, Iran; ^2^Neurocognitive Laboratory, Iranian National Center for Addiction Studies (INCAS), Tehran University of Medical Sciences (TUMS), Tehran, Iran; ^3^Students' Scientific Research Center (SSRC), Tehran University of Medical Sciences (TUMS), Tehran, Iran; ^4^School of Advanced Technologies in Medicine (SATiM), Tehran University of Medical Sciences (TUMS), Tehran, Iran; ^5^Head of Substance Abuse Prevention and Treatment Office (SAPTO), Mental Health, Social Health and Addiction Department (MeHSHAD), Ministry of Health and Medical Education (MoHME), Tehran, Iran; ^6^Translational Neuroscience Program, Institute for Cognitive Science Studies (ICSS), Tehran University of Medical Sciences (TUMS), Tehran, Iran

## Abstract

*Objective.* The aim of this study was to compare impulsivity and risky decision making among HIV-positive and negative heroin dependent persons.* Methods.* We compared different dimensions of impulsivity and risky decision making in two groups of 60 HIV-positive and 60 HIV-negative male heroin dependent persons. Each group was comprised of equal numbers of current (treatment seeker) and former (abstinent) heroin addicts. Data collection tools included Balloon Analogue Risk Task (BART), Iowa Gambling Task (IGT), Barratt Impulsiveness Scale (BIS), and Zuckerman Sensation Seeking Scale (SSS).* Results.* In SSS, comprised of four subscales including thrill and adventure seeking (TAS), experience seeking (ES), disinhibition (DIS), and boredom susceptibility (BS), there was a borderline difference in DIS (*P* = 0.08) as HIV-positive group scored higher than HIV-negative group. Also, ES and total score were significantly higher among HIV-positive patients. In BART, HIV-positive subjects scored higher in risk taking than HIV-negative subjects as reflected in higher Average Number of puffs in Successful Balloons (ANSB). In BIS, HIV-positive group scored significantly higher in cognitive impulsivity (CI) (*P* = 0.03) and nonplanning impulsivity (NPI) (*P* = 0.05) in comparison to HIV-negative group. Also, current heroin addicts scored significantly higher in NPI compared to former addict HIV-negative participants (*P* = 0.015). IGT did not show any significant difference between groups.* Conclusion.* Higher levels of impulsivity and risk taking behaviors among HIV-positive heroin addicts will increase serious concerns regarding HIV transmission from this group to other opiate dependents and healthy people.

## 1. Introduction

Impulsive behavior is defined as behavior that is performed with little or inadequate forethought and may lead to the performance of risky activities [1]. The possible predisposing role of impulsivity and sensation seeking has been previously addressed in the acquisition of HIV infection worldwide [2, 3]. On the other hand, psychological disorders and stigma in most of the People Living with HIV (PLWH) may lead to an increased rate of practicing impulsive and risky behaviors, that is, using nonsterile injection equipment and unprotected sexual activities [4]. Thus, the role of impulsive behavior is further highlighted with regard to the transmission of HIV infection from key populations at higher risk to the general population, especially in countries with concentrated epidemic [5].

The relationship between impulsivity and substance abuse is mutual; hence, substance abuse seems to be more prevalent among populations that score higher in impulsivity and sensation seeking measurement tools. In this regard, opiate abusers with higher impulsive behaviors are more likely to relapse after receiving appropriate treatments. Additionally, substance abuse may affect patients' cognitive and behavioral features. In fact, opiate abuse increases the practice of impulsive behavior which seems to be correlated with the severity of addiction. Additionally, impulsive drug users may show high levels of drug injection and unsterile needle use. Considering that using drugs through injection and unprotected sex are the major routes of HIV transmission, identification of the underlying psychological factors in HIV patients would be of utmost importance [6–8].

We conducted this study for the first time among Iranian PLWH to identify whether HIV infection is associated with impulsivity and sensation seeking as well as thrill and adventure seeking, experience seeking, boredom susceptibility, and nonplanning. Moreover, in order to elucidate the possible association of opiate addiction status (abstinence or active drug use) with impulsive behaviors, both HIV-positive and HIV-negative groups were comprised of equal numbers of current and former heroin addicts. Thus, the possible difference in risk taking, impulsivity rate, and sensation seeking between current and former heroin addicts could also be assessed.

## 2. Materials and Methods

### 2.1. Participants

This study was conducted among 120 participants who had a diagnosis of opiate dependence according to DSM-IV-TR criteria. The first group was comprised of 60 HIV-positive males who were recruited in Iranian Research Center for HIV/AIDS (IRCHA), Tehran, Iran. The second group included 60 HIV-negative men that were recruited in the Iranian National Center of Addiction Studies (INCAS), Tehran, Iran. Active heroin users were recruited from Methadone Maintenance Programs' (MMP) waiting lists and their heroin use was confirmed with positive urine analysis for morphine and clinical interviews. Former heroin users were recruited from MMP with at least six months' participation and negative urine tests during last month. All the participants were recruited from September 2008 to September 2009 with simple sequential nonrandom sampling and were in the same demographic conditions. They were between 18 to 50 years old and provided informed written consent prior to participation. Also, the Institutional Review Board of Tehran University of Medical Sciences reviewed and approved the study protocol. All the participants of both groups were evaluated with Enzyme-Linked Immunosorbent Assay (ELISA) test for HIV infection and positive results were confirmed with subsequent Western blot. AIDS-related dementia, CD4 < 500 cells/*μ*L, presence of any psychotic symptoms, diagnosis of dependence to other substances (except nicotine), history of head trauma, and presence of any medical conditions, that is, liver, renal, cardiovascular, and neurological diseases, were considered as the exclusion criteria.

### 2.2. Measurements

All of participants were assessed by demographic forms and four questionnaires by a professionally trained interviewer under similar environmental conditions. The demographic questionnaire included questions about age, level of education, and condition of opiate addiction. All of the questionnaires were previously validated and attested in terms of reliability [9].

(1) The Farsi version of Zuckerman Sensation Seeking Scale (SSS) which defines the underlying reason for making risky decisions as the need for experiencing varied and novel situations [9]: this questionnaire has 40 items which examine the individual differences in four subscales including thrill and adventure seeking (TAS), experience seeking (ES), disinhibition (DIS), and boredom susceptibility (BS). Each item includes 10 questions and is scored 0–10, while the sum of the scores would give the total score (0–40).

(2) The Farsi version of Balloon Analogue Risk Task (BART) test, which is a computerized model for measurement of risk-taking behavior [10]: in this test, participants are presented with 30 balloons on the computer screen which they may inflate by clicking the mouse button. Following each click, the balloon is either burst or further inflated, after which a specific amount of money is added to a temporary bank for that balloon. After each click, the participant may collect the temporary account at any point before the balloon bursts by clicking on a button marked as “collect” which would end the inflation of that balloon and add the temporary account to a safe account that is displayed on the screen. The more the eventual number of clicks for each balloon, the more money won from that balloon. The eventual size of balloons was variable and not predictable; hence, some balloons would reach the size of the screen while others would burst after several inflations. BART has a good correlation with the Iowa Gambling Task. The dependent measures of risk-taking in BART are defined as Average Number of puffs in Successful Balloons (ANSB), Number of Successful Balloons (NSB), and Average Number of puffs in Each Balloon (ANEB).

(3) The Farsi version of Barratt Impulsiveness Scale (BIS) which is a widely applied test for measurement of impulsivity [9]: the Farsi version of BIS used in this study was composed of 30 items on a four-point Likert scale focusing on the frequency of self-reported behavior (i.e., 1 = never/rarely and 4 = almost always) about three second-order factors including cognitive impulsivity (CI) (i.e., “I concentrate easily”), motor impulsivity (MI) (i.e., “I act on impulse”), and nonplanning impulsivity (NPI) (i.e., “I plan tasks carefully”). Thus, the items describe common impulsive behaviors and preferences about making decisions at special situations and thinking without relation to an exact time or location.

(4) The Farsi version of Iowa Gambling Task (IGT) in which participants are presented with four decks of cards labeled as A, B, C, and D [11]: for each deck of cards including 60 cards, a certain amount of reward and penalty has been considered. As the participant may see, the amount of reward and penalties per chosen card from decks A and B is more than cards from decks C and D. In this test, the participant should choose from four groups of decks in order to maximize the net amount of money won, while he/she is unaware of the total choices [12]. The net amounts of rewards and penalties are set in accordance with the fact that more choices from decks C and D would result in more amounts of total winning compared to the cards from decks A and B. After 100 choices are made, the total number of selections from advantageous decks (C and D) is subtracted from the number of selections from disadvantageous decks (A and B) and this difference is compared between the groups of study.

### 2.3. Statistical Analysis

Descriptive data were presented as mean ± SD and frequency for quantitative data. After performing the Kolmogorov-smirnov test of normality, we performed independent sample *t*-test to compare quantitative variables between the two groups. In order to compare the mean scores between the four study subgroups, one-way ANOVA and post hoc analysis (Tukey's honest significance test) were used. Afterward, we performed analysis of covariance (ANCOVA) to exclude the influence of covariates on the mean of scores that were significantly different between four study subgroups. SPSS version 17.0 was used for data analysis and *P* values equal to or less than 0.05 were considered statistically significant.

## 3. Results

### 3.1. Demographics and Substance Abuse History

One hundred and twenty male participants with the mean age of 38.5 ± 7.88 years and the educational average of 9.98 ± 2.43 years participated in this study. The total duration of opiate dependence was 9.90 ± 5.48 years. In the HIV-positive group, 31 participants were former heroin addicts and 29 of them were active heroin users. In the HIV-negative group, 30 participants were active heroin addicts and 30 were former addicts. Overall, 85 patients were crystalline heroin abusers (83 smokers and 2 injectors) among which 42 were HIV-positive and 42 were HIV-negative. Also, 35 patients were brown heroin abusers (29 heroin injectors, 3 heroin smokers, and 3 heroin sniffers) among which 18 were HIV-positive and 17 were HIV-negative. In the HIV-positive group, 17 patients were heroin injectors, while in the HIV-negative group, 12 patients were heroin injectors and 2 patients were crystalline heroin injectors. Based on analysis by age, education, and duration of addiction, there was no significant difference between the two groups of the study (Table 1).

### 3.2. The Zuckerman Sensation Seeking Scale (SSS)

Results of the performed measurements are presented in Tables 2 and 3. There was no significant difference in TAS and BS between the groups (Table 2); however, there was a borderline difference in DIS, as HIV-positive group scored higher compared to HIV-negative group (*P* value = 0.083). In the total score of this test, HIV-positive group scored significantly higher than HIV-negative group (*P* value = 0.03). Also, post hoc analysis showed that active opiate addict HIV-positive patients scored significantly higher than active opiate addict HIV-negative participants (*P* value = 0.007). Also, ES was significantly higher in active opiate addict HIV-positive patients compared to former addict HIV-negative participants (*P* value = 0.01). Additionally, HIV-positive patients with a positive history of Injection Drug Use (IDU) scored higher in ES (*P* value = 0.04) and DIS (*P* value = 0.01) compared to the HIV-negative group with a positive history of IDU, while the scores of TAS, BS, and total score were not significantly different between these two groups. In analysis of covariance of the ES score, result was correlated with the level of education (*P* value = 0.035). Even with the elimination of all factors, HIV infection had an effect on the ES scores (*P* value = 0.008, Table 4).

### 3.3. The Balloon Analogue Risk Task (BART)

HIV-positive group scored significantly higher than HIV-negative group regarding ANSB, NSB, and ANEB scores (Table 2). Also, analysis of the results between four groups showed significant difference too (*P* value = 0.024, Table 3). According to post hoc analysis, former addict HIV-positive patients scored significantly higher than former addict HIV-negative participants in all three parameters (*P* value = 0.015, *P* value = 0.001, and *P* value = 0.026 for ANSB, NSB, and ANEB, respectively). However, ANSB and ANEB were not significantly different between HIV-positive patients with a history of IDU and HIV-negative patients with a history of IDU, while the difference in NSB was borderline significant (*P* value = 0.07). There was no significant relationship between the items of this test and age, years of education, and addiction duration as demographic variables (Table 4).

### 3.4. The Barratt Impulsiveness Scale (BIS)

After the examination of all three subscales between participants, HIV-positive group scored higher than HIV-negative group in CI and NPI (Table 2). Although this difference was significant for CI (*P* value = 0.035), it was borderline significant for NPI (*P* value = 0.053). Between four subgroups of the study, there was a significant difference in CI (*P* value = 0.033) and NPI (*P* value = 0.018, Table 3). Post hoc analysis showed that active addict HIV-positive patients scored higher in NPI compared to former addict HIV-negative participants (*P* value = 0.015). Also, former addict HIV-positive patients scored higher in CI compared to current addict HIV-negative group (*P* value = 0.020). Moreover, HIV-positive patients with a history of IDU scored higher compared to HIV-negative patients with a history of IDU in MI (*P* value = 0.02) and total BIS score (*P* value = 0.02), while the differences in NPI and CI were borderline significant (*P* value = 0.07 and *P* value = 0.07, respectively). NPI had a significant relationship with age (*P* value = 0.014). CI was also significantly related to age (*P* value = 0.014), education (*P* value = 0.003), and addiction duration (*P* value = 0.015, Table 4).

### 3.5. The Iowa Gambling Task (IGT)

There was no significant difference between HIV-positive group and HIV-negative group in three variables of total duration of IGT (seconds), net amount of win (win − loss), and IGT total score. Also the results did not reveal any significant difference among the four groups of the study. HIV-positive patients with a history of IDU did not score significantly different compared to HIV-negative patients with a history of IDU in any of the three variables of IGT.

## 4. Discussion

In the current study, we used multiple tests to assess the differences in impulsive behaviors, sensation seeking, and risk-taking among HIV-positive and negative heroin addicts. Hence, both groups were comprised of current and former addicts, and the relationships of current addiction status with each of the measured behavioral characteristics were also evaluated. According to our findings, HIV-positive opiate dependent persons reported higher levels of disinhibition in comparison with HIV-negative opiate dependent persons, regardless of addiction status, which implies that they may engage more in disinhibited behaviors that could enhance the risk of HIV transmission. Also, our results indicated that HIV patients may perform more risky behaviors due to their higher score in experience seeking which is in line with the findings of Hardy et al. [5]. As a matter of fact, HIV infection has reached concentrated epidemics among Iranian people who inject drugs; accordingly, the practice of impulsive behaviors and more experience seeking among this group (i.e., unprotected sexual intercourse) may further result in the spread of infection [13, 14]. In line with this explanation, Gullette and Lyons declared that the scores of SSS are associated with the practice of risky behaviors such as unprotected sex with commercial sex workers, which may lead to the transmission of HIV epidemic from HIV-positive drug addicts to other key populations [15].

In our findings regarding comparison of BART, HIV-positive group scored higher than HIV-negative group in the three subscales which are known to be related to more risk-taking behaviors. This is in line with previous reports that showed HIV infection is related to more risk-taking [16], although the definite influence of HIV infection on risk-taking behavior remains unidentified. In fact, the current literature lacks enough evidence to support the idea that the incidence of HIV infection among opiate users with more risk-taking behavior is more compared to opiate users who have scored less in such tests. Additionally, modeling of the participants' behavior in BART has revealed that poor performance in heroin abusers is probably due to reward-dependency and insensitivity to evaluation [16, 17]. Furthermore, the results of BIS in our survey showed that HIV infected patients scored higher with regard to nonplanning impulsivity and cognitive impulsivity in comparison with HIV-negative group.

With regard to opiate addiction, the differences between active drug users and former users engaging in impulsive behaviors corroborate the result of Passetti et al. [18]. Their finding was related to the fact that addiction can influence decision-making and increase the performance of impulsive behaviors [19–21]. Previously, Stout et al. used cognitive modeling to study decision-making deficits in cocaine-abusers. Their study showed that cocaine-abusers have poor performance in IGT [22]. In fact, HIV infected substance users may have impaired performance on measures that involve the prefrontal-subcortical systems such as the IGT [23]. In our analysis, however, there was no significant difference in IGT results between HIV-positive group and HIV-negative group. Such observed differences could be explained by the different social settings as well as the diversity of attitudes and knowledge about stigmatization toward addiction and HIV infection between societies [24, 25]. The absence of group differences in IGT in our study may also be explained by the fact that there are different behavioral traits of impulsivity. Thereby, although we were not able to identify a significant difference in the results of IGT concerning cognitive impulsivity, the HIV infected patients scored higher in the cognitive impulsivity subscale measured by BIS. This finding may indicate a higher level of self-reported cognitive impulsivity compared to impulsive decision-making among HIV-positive patients. Although the relevance of observed differences in the obtained scores of self-reported cognitive impulsivity remains unidentified in this study, it may be related to the high levels of perceived stigma among Iranian PLWH [26].

This study has several limitations: first, we did not assess the effect of self-reported risky behaviors such as injection history, risky and unprotected sexual behaviors, aggression, and history of imprisonment on the measured items. Also, we were unable to collect data regarding the route of HIV acquisition and duration of infection among HIV-positive group. Another limitation was that all of our participants were male subjects. The sample size of our study was relatively small. For future studies, we suggest investigation of the risk-taking and impulsivity among HIV-positive active drug users in female subjects and other key populations, that is, sex workers.

In conclusion, heroin dependent PLWH of this study scored significantly higher than HIV-negative group on different risk taking, impulsivity, and sensation seeking dimensions, which may render them susceptible to the practice of more risky behaviors. Such risky behaviors may further decrease the adherence to antiretroviral therapy and negatively impact their social relationships. Another consequence of practicing risky behaviors among opiate dependent HIV patients is the possible contribution of such behaviors to the spread of HIV to the general public. Thereby, interventions such as behavioral educations or psychosocial rehabilitations should focus on decreasing the practice of impulsive behaviors among this group of patients. Since the prevalence of psychiatric disorders is quite high among PLWH [27, 28], the timely diagnosis of substance abuse and concomitant psychological impairments for performing interventions that would halt the progress of perceived stigmatization is of great significance. Also, teaching PLWH to use coping strategies to deal with HIV stigma may lead to the improvement of the overall psychological well-being [29]. Considering that the main route of HIV acquisition in Iran is using drug and that current heroin addicts in both groups scored higher in NPI compared to former addicts, preventive and therapeutic interventions aiming at opiate addiction may further decrease the rate of HIV transmission in Iran.

## Figures and Tables

**Table 1:** Age, education, and duration of heroin addiction among HIV-positive and HIV-negative participants of the study.

Variable^*∗*^	HIV-positive group	HIV-negative group	*P* value^♦^
Age (years)	36.43 ± 7.65	35.27 ± 8.13	0.42
Education (years)	9.83 ± 2.56	10.13 ± 2.31	0.50
Heroin addiction duration (years)	10.15 ± 4.17	9.64 ± 6.57	0.61

^*∗*^Data are represented as mean ± SD

^♦^Independent sample *t*-test.

**Table 2:** Comparison of subscale scores in each test between HIV-positive group and HIV-negative group.

Item^*∗*^	HIV-positive group	HIV-negative group	*P* value^♦^
Zuckerman Sensation Seeking Scale (SSS)			
Thrill and adventure seeking	6.70 ± 2.80	6.33 ± 2.55	0.45
Experience seeking	4.28 ± 1.90	3.30 ± 2.03	0.007
Disinhibition	3.52 ± 1.65	2.95 ± 1.89	0.083
Boredom susceptibility	2.70 ± 1.65	2.48 ± 1.44	0.44
SSS total score	17.20 ± 5.75	15.07 ± 5.13	0.03

Balloon Analogue Risk Task (BART)			
Average Number of puffs in Successful Balloons	34.58 ± 17.11	25.15 ± 13.35	0.002
Number of Successful Balloons	17.52 ± 4.02	22.63 ± 4.00	<0.001
Average Number of puffs in Each Balloon	30.40 ± 12.40	23.64 ± 11.58	0.004

Barratt Impulsiveness Scale (BIS)			
Nonplanning impulsivity	28.38 ± 4.10	26.75 ± 4.99	0.053
Motor impulsivity	24.43 ± 5.04	24.57 ± 6.51	0.9
Cognitive impulsivity	19.47 ± 4.27	17.67 ± 4.94	0.035
BIS total score	72.25 ± 10.67	69.08 ± 14.02	0.16

Iowa Gambling Task (IGT)			
Total duration of IGT (seconds)	286.54 ± 168.38	314.54 ± 144.77	0.38
Net amount of win (win − loss)	−12667.50 ± −11223.32	−14242.93 ± 14446.33	0.55
IGT total score [(*C* + *D*)−(*A* + *B*)]	−2.56 ± 21.54	−5.65 ± 29.38	0.55

^*∗*^Data are represented as mean ± SD.

^♦^Independent sample *t*-test.

**Table 3:** Comparison of subscale scores in each test between current and former opiate addict HIV-positive and HIV-negative participants.

Item^*∗*^	HIV-positive group		HIV-negative group		*P* value^♦^
	Current addicts	Former addicts	Current addicts	Former addicts	
Zuckerman Sensation Seeking Scale (SSS)					
Thrill and adventure seeking	6.41 ± 2.99	6.97 ± 2.63	6.33 ± 2.18	6.33 ± 2.91	0.75
Experience seeking	4.93 ± 2.08	3.68 ± 1.51	3.27 ± 1.72	3.33 ± 2.33	0.004
Disinhibition	3.72 ± 1.85	3.32 ± 1.44	2.77 ± 1.99	3.13 ± 1.79	0.22
Boredom susceptibility	3.07 ± 1.79	2.35 ± 1.45	2.73 ± 1.23	2.23 ± 1.61	0.14
SSS total score	18.14 ± 6.31	16.32 ± 5.12	15.10 ± 4.59	15.03 ± 5.70	0.10

Balloon Analogue Risk Task (BART)					
Average Number of puffs in Successful Balloons	33.00 ± 13.57	35.93 ± 19.80	26.66 ± 14.78	23.70 ± 11.88	0.012
Number of Successful Balloons	17.63 ± 3.63	17.43 ± 4.40	22.21 ± 4.25	23.03 ± 3.77	<0.001
Average Number of puffs in Each Balloon	29.21 ± 10.12	31.43 ± 14.18	24.93 ± 12.82	22.40 ± 10.32	0.02

Barratt Impulsiveness Scale (BIS)					
Nonplanning impulsivity	29.83 ± 4.08	27.03 ± 3.70	27.23 ± 5.57	26.27 ± 4.38	0.018
Motor impulsivity	27.07 ± 4.58	23.84 ± 5.45	25.30 ± 6.30	23.83 ± 6.84	0.64
Cognitive impulsivity	20.59 ± 4.13	18.42 ± 4.19	17.07 ± 4.09	18.27 ± 5.68	0.033
BIS total score	75.41 ± 9.77	69.29 ± 10.77	69.60 ± 13.45	68.57 ± 14.79	0.13

Iowa Gambling Task (IGT)					
Total duration of IGT (seconds)	268473.43 ± 104624.35	301947.19 ± 208890.0	333373.52 ± 185294.59	296714.35 ± 88304	0.58
Net amount of win (win − loss)	−13141.30 ± 10152.75	−12263.89 ± 12239.43	−14526.09 ± 12693.19	−13959.78 ± 16297	0.93
IGT total score [(*C* + *D*)−(*A* + *B*)]	−3.48 ± 18.29	−1.78 ± 24.30	−5.57 ± 26.47	−5.74 ± 32.63	0.94

^*∗*^Data are represented as mean ± SD.

^♦^One-way ANOVA.

**Table 4:** Models of the covariance analysis (ANCOVA) for exclusion of the influence of covariates were obtained for experience seeking domain of the sensation seeking scale, variables of the balloon analogue risk task (Average Number of puffs in Successful Balloons, Number of Successful Balloons, and Average Number of puffs in Each Balloon), and the nonplanning and cognitive impulsivity from Barratt Impulsiveness Scale.

	Corrected model	Intercept	Age (years)	Education (years)	Opiate dependence duration (years)	HIV infection	Opiate dependence status (former versus current)	Receiving HIV treatment
Experience seeking								
Mean square	14.27	107.10	7.22	16.25	11.00	25.83	5.53	7.69
*F*	3.98	29.87	2.01	4.53	3.06	7.20	1.54	2.14
*P* value	0.001	<0.01	0.159	0.35	0.083	0.008	0.217	0.146

Average Number of puffs in Successful Balloons								
Mean square	527.04	4193.4	174.28	15.69	47.10	2487.03	14.19	179.10
*F*	2.21	17.58	0.731	0.066	0.197	10.42	0.018	0.751
*P* value	0.048	<0.01	0.395	0.798	0.658	0.002	0.895	0.388

Number of Successful Balloons								
Mean square	126.94	847.47	1.09	5.28	15.91	720.09	1.21	2.75
*F*	7.63	50.95	0.066	0.318	0.957	43.29	0.73	0.165
*P* value	<0.01	<0.01	0.798	0.574	0.330	<0.01	0.787	0.685

Average Number of puffs in Each Balloon								
Mean square	281.03	3064.51	153.83	0.058	4.14	1284.11	0.448	135.18
*F*	1.90	20.74	1.04	<0.01	0.028	8.69	0.003	0.915
*P* value	0.87	<0.01	0.310	0.984	0.867	0.004	0.956	0.341

Nonplanning impulsivity								
Mean square	68.76	3533.44	118.46	55.24	29.42	91.09	75.97	11.19
*F*	3.64	187.10	6.27	2.92	1.55	4.82	4.02	0.593
*P* value	0.002	<0.01	0.014	0.09	0.215	0.03	0.047	0.443

Cognitive impulsivity								
Mean square	93.89	2113.70	114.29	172.64	112.28	86.46	0.006	40.24
*F*	5.13	115.58	6.25	9.44	6.14	4.72	<0.01	2.20
*P* value	<0.01	<0.01	0.014	0.003	0.015	0.032	0.986	0.141
